# *Integrase-On-Demand*: bioprospecting integrases for targeted genomic insertion of genetic cargo

**DOI:** 10.1093/nar/gkag106

**Published:** 2026-02-09

**Authors:** Hannah M McClain, Lillian C Lowrey, Laura B Quinto, Ellis L Torrance, Tomas R Gagliano, Farren J Isaacs, Joseph S Schoeniger, Kelly P Williams, Catherine M Mageeney

**Affiliations:** Systems Biology Department, Sandia National Laboratories, Livermore, CA 94550, United States; Biotechnology and Bioengineering, Sandia National Laboratories, Livermore, CA 94550, United States; Department of Molecular, Cellular and Developmental Biology, Yale University, New Haven, CT 06511, United States; Systems Biology Institute, Yale University, West Haven, CT 06516, United States; Systems Biology Department, Sandia National Laboratories, Livermore, CA 94550, United States; Biotechnology and Bioengineering, Sandia National Laboratories, Livermore, CA 94550, United States; Department of Molecular, Cellular and Developmental Biology, Yale University, New Haven, CT 06511, United States; Systems Biology Institute, Yale University, West Haven, CT 06516, United States; Department of Biomedical Engineering, Yale University, New Haven, CT 06511, United States; Biological, Radiation, and Signature Science, Technology, and Engineering Center, Sandia National Laboratories, Livermore, CA 94550, United States; Systems Biology Department, Sandia National Laboratories, Livermore, CA 94550, United States; Biotechnology and Bioengineering, Sandia National Laboratories, Livermore, CA 94550, United States

## Abstract

Integrases serve as powerful biotechnology tools that catalyze recombination at specific DNA sequences (*att* sites) and facilitate chromosomal integration of gene cargos transferred into cells. Given that genomes often lack the *attB* integration sites recognized by frequently utilized integrases, integrase technology has largely been restricted to genetic engineering of model organisms into which *attB* sites can be synthetically introduced. To enable single-step site-specific integrase-mediated genome editing in a broad spectrum of prokaryotes, we have devised the *Integrase-On-Demand* (IOD) method. IOD systematically identifies integrases, within bacteria and archaea, that can integrate into available *attB* sites in any target prokaryote. Computational results show that diverse bacteria generally have multiple potentially useable native *attB* sites for novel integrases. We confirmed the functionality of predicted integrase and *attB* pairs for mediating site-specific integration of heterologous DNA into the genomes of *Pseudomonas putida* S12 and KT2440 and *Synechococcus elongatus* UTEX 2973, measuring efficiency of integration using nonreplicating vectors. By eliminating the requirement to introduce non-native *attB* sites into the target genome, IOD may, when suitable transformation methods exist, allow facile genome integration of large constructs in nonmodel and possibly nonculturable bacteria.

## Graphical Abstract

**Graphical Abstract:**
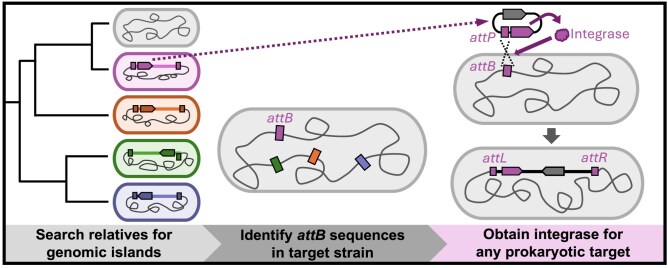

## Introduction

Genome engineering enables sophisticated genetic and functional genomic studies in bacteria and microbiomes. Moreover, it drives advances in biotechnology applications, benefiting biomanufacturing [1], bioremediation [2], soil health and rhizosphere adaptation [3], and human health [4]. As biotechnology applications have diversified, and research into microbial ecosystem enabled by metagenomics has expanded, less well-studied, genetically and metabolically diverse microbes have elicited interest. These organisms often lack established genetic tools, or may be difficult to isolate or culture, which impedes the use of standard genetic tools for research and synthetic biology efforts to introduce novel or enhanced functions. Such concerns are particularly acute for applications such as, genetic engineering of specific members of microbial communities or microbiomes.

Mobile integrative genetic elements are delivered by phage particles or conjugation pili into bacterial or archaeal cells and integrate within the genome of the host as a genomic island (GI). Because of their foreign nature and mobility, GIs typically contain sequences which are not found in the host core genome but which are shared sporadic among strains of a species [5]. GIs often encode integrases that integrate their incoming DNA within the host chromosome. Integrases catalyze DNA recombination between attachment (*att*) sites in the mobile element (*attP*) and in the chromosome (*attB*) [6]. These *attB* sites are typically found within tRNA and tmRNA genes or intergenic sequences (56.6% of GIs from our recent TIGER database [7]), though many are found in protein-coding genes [5]. In many cases, Gis, unlike transposons, reconstitute the genes they insert within to preserve function [7–9]. However, it is possible that GI integration may lower host fitness. Recombination produces two hybrid *att* sites, *attL* and *attR*, that flank the GI within the chromosome, precisely defining its ends, and serving as substrates for a potential GI excision reaction that generally requires both the integrase and a Recombination Directionality Factor or excisionase (*xis*) gene [10]. Two unrelated integrase families exist, tyrosine and serine recombinases. While serine integrases are more commonly utilized in biotechnology, both families are capable of catalyzing integration, inversion, and excision of precisely defined regions of DNA.

Integrases provide a potential solution for efficiently introducing large genetic cassettes into a range of prokaryotes [11, 12]. They have many advantages over other methods of genetic engineering including insertion of large DNA payloads [12], scarless insertions [6], conservation of *attB* sites between related taxa [9], and site-specific insertion with minimal off-target activity [13]. Technologies have been developed to utilize integrases for genome engineering [14], including programmable addition via site-specific targeting elements (PASTE) [15], recombinase mediated cassette exchange (RMCE) [16], and serine recombinase-assisted genome engineering (SAGE) [17]. However, these methods typically require two transformation and selection steps. The first introduces non-native *att* sites as a ‘landing pad’ (i.e. integration site) for subsequent DNA cargo integration (e.g. using random, highly mutagenic transposon insertion) and the second delivers the integrase and associated cargo genes into the randomly placed *att* site. Transformation, especially for nonmodel bacteria and archaea, can be very inefficient, and in some scenarios, such as engineering a nonisolatable/nonculturable member of a microbiome, multiple transformation and selection steps are either not feasible or would simply destroy the community being studied [18–20].

To facilitate site-specific genomic insertion of large genetic cargo in a wide range of prokaryotes, including members of communities, we have developed the *Integrase-On-Demand* (IOD) method. Recent advances in GI discovery, enabled by our Islander and TIGER software, have greatly expanded the association of tyrosine and serine integrases with their precisely predicted *att* sites across hundreds of thousands of diverse prokaryotic genomes [20]. IOD starts with a computational pipeline that leverages these advances to identify prokaryotic serine and tyrosine integrases predicted to recognize *attB* sequences present within a user-defined bacterial or archaeal strain of interest. Here, we demonstrate that the IOD software can predict integrases and *att* sequences from diverse species. As proof of concept for the IOD method, we experimentally validated that nonreplicating vectors could insert site-specifically, efficiently, and stably into the chromosomes of two phylogenetically distant and biotechnologically relevant bacterial species: the gammaproteobacterium *Pseudomonas putida* [strains S12 (nine integrases) and KT2440 (three integrases)], and the cyanobacterium *Synechococcus elongatus* UTEX 2973 (one integrase).

## Materials and methods

### Development of IOD software

**Figure 1.**
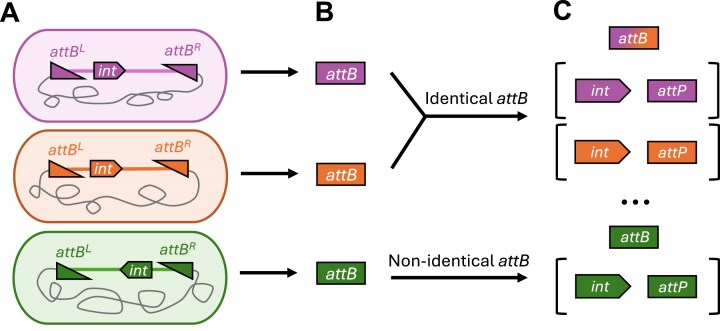
Preprocessing steps for IOD Integrase and *att* pairs. **(A)*** attB* sites are extrapolated from TIGER/Islander predicted GIs. **(B)**  *attB*-integrase pairs are built based on *attB* sequence identity. **(C)** At least one integrase and predicted GI is associated with each *attB* sequence.

IOD software references a database of *attB* sequences and their corresponding integrase proteins to provide the user with a list of candidates for use in a target genome. Each *attB*/integrase pair is derived from a GI predicted by the TIGER/Islander programs [5, 8, 21]. These programs provide detailed information on the predicted GIs, such as the sequences of the recombinant sites, *attB* sequence, and identifiers for integrases encoded within the putative GI. The programs have been run on over 450,000 genomes, yielding 1,757,053 GIs containing integrases paired with their cognate *att* sites [7]. To build the reference data structure, the set of unique *attB* sequences of the predicted GIs was collected (Fig. 1).

Integrases theorized to be inactive due to truncation or lack of catalytic domains were excluded. To assess tyrosine integrase integrity, we compared these integrases to the 20 HMMs of the tyrosine recombinase database [22]. Putative tyrosine integrases with no HMM match, or that matched to the Xer or Integron HMMs (subfamilies that perform nonintegrase functions) were rejected. Furthermore, tyrosine integrases longer than 800 amino acids were rejected. Finally, a metric (∆∆HA) was developed to identify truncated proteins, calculating the number of amino acid positions of the HMM missing at the termini of the protein (∆H) and the number of amino acids at the termini of the protein exceeding the HMM hit (∆A), and taking ∆∆HA = ∆H - ∆A. We used a cutoff of ∆∆HA > 100, which passed 98.5% of the medium-sized tyrosine integrases (250–800 aa) and 8.9% of the short tyrosine integrases (<250 aa), to reject nominally truncated proteins. Lastly, serine integrases were rejected unless: (i) the integrase had the Pfam Resolvase domain followed by the Pfam Recombinase domain and was not too long (>800 amino acids) or too short (∆∆HA > 0); or (ii) our Tater software had previously classified it as S-Core_IS607 and it was not too long (>300 aa) or too short (∆∆HA > 0). As a result, the final list of *attB* sites queried by IOD is 182 734 unique sequences.

The IOD program features two modes (*search* and *taxonomic*) along with several command line options. *Search* mode can be invoked to search any sequence against the genome of interest. The user may provide specific *attB* sequences to search or use the entire de-duplicated *attB* database (182 734). If a match is found in the genome of interest, the *attB* site, as well as the sequence(s) of the cognate integrase(s), are returned to the user. *Taxonomic* mode searches for *attB* sites found in close relatives to the genome of interest. Users can modify the minimum number of *attB* sequences to collect in *taxonomic* mode prior to searching (default: *n* = 500), length of *attB* flanks from serine (default: sl = 16), or tyrosine (default: yl = 10) integrases, and number of threads for BLAST to use (default: threads = 1).

*Taxonomic* mode runs MASH [23], a program that uses a hash function to reduce genomic sequences to representative sketches. A sketch of the user-provided genome is created and compared to a pre-compiled database of 85 205 Genome Taxonomy Database (GTDB) representative species [24] and returns a list of genomes which have high similarity to the query. Starting with the best candidate species, the *attB* sequences and their respective integrase(s) from within the query species are collected, reaching out to other species or genera, as needed, to reach the desired minimum number (default: *n* = 500).

A query FASTA including each full reference *attB*, and its two half sites *attB ^L^* (left flank and identity block of *attB*), and *attB^R^* (identity block and right flank of *attB*) is prepared. The half sites are used to probe for GIs occupying *attB*s against a nucleotide BLAST database is produced from the user-provided genome [25] using the flag *-task blastn*. Hits are then rejected if they have (i)< 95% identity, (ii) more than one gap in the alignment, (iii) more than two mismatches, or (iv) any combination of two or more hits to the same full *attB* or its half sites in the genome. Hits rejected in the final filter are retained in a separate list of possible GI-occupied *attB*s. To characterize a GI-occupied site, the length of each reference GI utilizing the *attB* is collected from the database to determine which GI could be present. Each GI size is then compared to the distance between the *attB^L^* and *attB^R^* hits. If the *attB* halves map to different contigs, then the direction of the BLAST hit is used to determine which contig termini would be expected to be part of a GI. To be predicted as an occupied site, the normalized (for length of expected GI) difference must be <0.05:


\begin{eqnarray*}
{\mathrm{ normalized}}\ {\mathrm{ difference}} = \frac{{{\mathrm{ expected}} - {\mathrm{ actual}}}}{{{\mathrm{ expected}} + {\mathrm{ actual}}}}
\end{eqnarray*}

Following the categorization of all the BLAST hits, the candidates are passed through a final refining filter to remove questionable *attB*s. These are defined as *attB*s found within occupied sites, *attB*s with multiple copies of the ID-block (bioinformatic-based estimate of the crossover site) elsewhere in the genome, or *attB*s with single-nucleotide tandem repeats in the ID-block.

Despite deduplication of sequences in pre-processing, the list of candidates often contains overlapping *attB* sites. Shifted *attB* sites could be explained by mutations between closely related species that share a GI and/or the limit of precision in integration sites called by TIGER/Islander software [5, 8, 21]. The program bins all candidates by overlapping coordinates and attempts to select the highest quality sequence from each bin according to the best BLAST support score. If there are ties, then the site whose reference GI has the highest support will be selected. The best candidate *attBs* are reported in *final_candidates.tsv* in ranked order according to the best score value of the reference GI(s) from which the *attB* was sourced. Each candidate site reports an example reference GI according to the highest support. If an *attB* has more than one reference GI, the final column will be populated with the GI ID (GCA accession and gene ID) and integrase for each reference. All candidates, including questionable candidates removed by the refining filter and deduplicated sites are reported in *attb_dupes.tsv*. Additionally, all *attB* sites are reported with each associated reference GI in isles*.json*. Similarly, occupied sites are deduplicated by overlapping coordinates and reported in *occupied.tsv* along with the reference GI.

### Software testing

Two sets of genomes were collected for study. The first set (Health) contained 41 genomes of health and biomanufacturing relevance. The second set (Diversity) contained 142 genomes each representing a different GTDB phylum with >4 genomes available.

Both genome sets were tested in *taxonomic* and *search* modes using default parameters (Table 1 and Supplementary Table 1). To simulate a typical user experience, the software was tested locally on a MacBook Air, M15,13. IOD was written in python (v3.12) and requires the external programs MASH (v2.3) (https://github.com/marbl/Mash) and the BLAST (v2.16) search algorithm to be available as a system-wide executable. The program can be run from any command line interface. Memory consumption was found to peak at 14.6 MB and 14.4 MB, for *taxonomic* and *search* mode, respectively (Supplementary Fig. 1). The software and README is available at https://github.com/sandialabs/Integrase-On-Demand.

**Table 1.** Taxonomic mode IOD output summary for the Health genome set of 41 organisms relevant for human health or biomanufacturing

GTDB species	GCA	*attB* queries	*attB* candidates	*attB*s in transfer RNA (tRNA) genes	Occupied
***Bacillus coagulans* MA-13**	004359975	500	3	2	0
***Corynebacterium glutamicum* ATCC13032**	000011325	152	10	7	2
***Bacteroides fragilis***	000025985	500	38	12	10
***Corynebacterium diphtheriae***	001457455	203	16	7	3
***Acinetobacter baumannii***	009759685	500	34	10	8
***Clostridioides difficile* DSM28196**	003482035	500	28	0	28
***Clostridium tyrobutyricum* ATCC25755**	000359585	286	8	3	9
***Pseudomonas aeruginosa***	001457615	500	46	15	6
***Zymomonas mobilis* ZM4**	003054575	111	0	0	0
***Pseudomonas putida* S12**	000495455	500	37	15	7
***Clostridium ljungdahlii* DSM13528**	000143685	214	1	0	5
***Sulfololobus acidocaldarius***	000012285	33	0	0	0
***Streptomyces venezualae***	008639165	500	20	10	16
***Rhodobacter sphaeroides* ATCC17023-2.4.1**	000012905	500	9	6	10
***Pseudomonas putida* KT2440**	000007565	500	32	12	11
***Novosphingobium aromaticivorans* DSM12444**	000013325	500	10	8	4
***Haloferax volcanii***	000025685	500	10	5	12
***Clostridium acetobutylicum* ATCC824**	000008765	500	0	0	1
***Vibrio cholerae***	000621645	500	27	10	3
***Streptomyces griseus* ATCC13273**	003610995	500	30	15	7
***Methylobacterium aquaticum***	001043915	500	6	6	5
***Synechococcus elongatus***	000817325	72	1	1	0
***Methylorubrum extorquens***	900234795	500	23	20	6
***Escherichia coli***	003697165	500	44	8	8
***Enterococcus faecalis***	000392875	500	38	5	11
***Klebsiella pneumoniae***	000742135	500	37	9	11
***Bacillus subtilis***	000009045	500	16	2	3
***Bacillus licheniformis DSM13***	000011645	500	24	9	2
***Helicobacter pylori***	900478295	500	22	1	0
***Bacillus coagulans* DSM2314**	006716385	482	4	2	0
***Neisseria gonorrhoeae***	003315235	500	17	12	10
***Pseudomonas fluorescens* SBW25**	000009225	500	29	16	10
***Listeria monocytogenes***	900187225	500	38	7	0
***Cupriavidus necator* H16**	004798725	500	27	17	5
***Acidithiobacillus ferrooxidans***	000021485	293	24	18	2
***Streptococcus pneumoniae***	001457635	500	43	3	15
***Leptospirillum ferriphilum***	000755505	67	11	11	3
***Staphylococcus aureus***	001027105	500	25	2	6
***Salmonella enterica***	000006945	500	34	9	9
***Mycobacterium tuberculosis***	000195955	500	24	16	5
***Francisella tularensis***	000008985	500	3	2	0

GTDB species name, the NCBI genome assembly accession number, number of *attB* sequences used, number of *attB* candidates, subset of *attB* candidates sourced from GIs targeting tRNA genes, and number of occupied sites are listed. Supplementary Table 1 has the full data set with search mode results included.

### Phylogenetic analysis

The protein sequence for each integrase predicted by the IOD software was obtained. Serine and tyrosine integrase sequences were treated separately. Protein sequences were aligned using MAFFT v7.526 [26] (mafft –auto) and maximum-likelihood trees were prepared using RAxML-NG v1.2.2 [27] (raxml-ng –all –msa tyr.afa –model LG + G8 + F –tree pars 10 –bs-trees 200). The resultant phylogenetic tree was viewed using FigTree v1.4.4 (http://tree.bio.ed.ac.uk/software/figtree/) and rooted at the midpoint.

### Protein alignments for catalytic residue confirmation

We used the same method as described above to create the aligned protein sequence separately for the serine integrases and tyrosine integrases. For the serine integrases we obtained the integrase reference sequences from Uniprot [28] using the following accession numbers: PhiC31 (Q9T221) and Bxb1 (Q9B086). We obtained Lambda (P03700) and Cre (P06956) as model tyrosine integrases. The aligned protein sequences were loaded into the SnapGene viewer to create the final figure and confirm catalytic residue positions.

### GI prediction, induction, and recombination measurements

TIGER [5, 21] and Islander [8] were used to predict GIs in *P. putida* S12 (assembly: GCF_000495455.2).

*Pseudomonas putida* S12 was grown overnight at 30°C shaking in Luria broth (LB), diluted 3:100, and grown to an OD_600_ of 0.4 and 1 μg/ml of mitomycin C (MMC) was added. Samples (1 ml) were collected at 0, 1, 2, and 3 h post MMC addition. Cells were pelleted at 16000 × *g* for 2 min and genomic DNA was isolated using the Qiagen DNeasy blood and tissue kit (Qiagen, Venlo, Netherlands, 69506). Genomic DNA libraries were prepared using the Illumina DNA prep kit (Illumina, San Diego, CA, 20060060; Indexes: Illumina, San Diego, CA, 20018708). Libraries were pooled in eqi-molar fashion, and the final library was sequenced using an Illumina NextSeq 500 sequencer with the high-output 300-bp single-end read sequencing kit (Illumina, San Diego, CA, 20024908).

Sequencing reads were quality filtered using BBDuk (v36.11; http://jgi.doe.gov/data-and-tools/bb-tools/) with the following parameters specified: ktrim = r, k = 21, mink = 11, hdist = 1. The filtered reads were analyzed with Juxtaposer [29] to identify noncanonical reads compared to the *P. putida* S12 reference genome. The resultant *juxtas.txt* file was used to create a graph of recombinant reads by placing the identified noncanonical read into a single 500-bp bin across the genome.

### Bacterial strains and culturing

Cyanobacterium *S. elongatus* UTEX 2973 was cultured under 100 or 200 µE white light at 38°C with 0.5% CO_2_ in 1× BG-11 medium (prepared from Sigma–Aldrich, Darmstadt, Germany, C3061-500ML). Donor (Ec100D pir + cloning strains) and helper (HB101 with conjugal plasmid pRL443) *Escherichia coli* strains for cyanobacterial conjugation were cultured at 30°C in LB Lennox medium (Sigma, L3022-6 × 1KG). NEB 5-alpha *E. coli* (NEB, Ipswich, MA, C2987H), NEB 5-alpha F’I^q^*E. coli* (NEB, Ipswich, MA, C2992I), and *P. putida* were cultured in LB medium (Sigma–Aldrich, Darmstadt, Germany, L3522) at 37°C (*E. coli*) or 30°C (*P. putida*). When applicable, media were supplemented with 100 µg/ml diaminopimelic acid (DAP; Fisher AAB2239106) (*E. coli*), 50 µg/ml carbenicillin (Sigma C1389-5G; *E. coli*), 34 µg/ml of chloramphenicol (Teknova, Hollister, CA, C0322) (*E. coli*), 50 µg/ml kanamycin sulfate (American Bio, AB01100-00010; *S. elongatus*), or tetracycline (Teknova, Hollister, CA, T3325) at 10 µg/ml (*E. coli*) or 25 µg/ml (*P. putida*).

### Plasmid construction

To generate plasmids for integrase testing in *P. putida*, DNA sequences consisting of a 600 bp *attP* sequence (composed of the computationally determined *att* identity block flanked by GI DNA), a *tac* promoter, and the corresponding *int* were synthesized by Twist Biosciences (South San Francisco, CA). These sequences were then cloned into pACYC184 bearing additional I-SceI sites using NEBuilder HiFi Assembly mix (NEB, Ipswich, MA, E2621L). Integrase-testing plasmids were maintained with chloramphenicol selection in NEB 5-alpha F’I^q^*E. coli*. Plasmids encoding frameshifted integrases were generated through either error-prone DNA synthesis or using inverse polymerase chain reaction (PCR) to delete a nucleotide within the *int* sequence. Plasmids with mutant *attP* sequences contained 600 bp false *attP* sequences from *att*-flanking non-GI DNA.

To generate the positive control plasmid pUCP22-*tcR, tcR* (conferring tetracycline resistance) from pACYC184 was PCR amplified with Q5 DNA polymerase (NEB, Ipswich, MA, M0491S) and cloned into pUCP22 using restriction cloning (Eco53kI (NEB, Ipswich, MA, R0116S), XbaI (NEB, Ipswich, MA, R0145S), Monarch^®^ Spin PCR & DNA Cleanup Kit (NEB, Ipswich, MA, T1130L), Ligase (NEB, Ipswich, MA, M0202L). pUCP22-*tcR* was maintained in NEB 5-alpha *E. coli* with tetracycline selection. To verify all plasmid sequences, whole plasmid sequencing was performed by Plasmidsaurus (Louisville, KY) using Oxford Nanopore Technology with custom analysis and annotation.

Plasmids for integrase testing in *S. elongatus* UTEX 2973 were generated using Gibson assembly. Constituent fragments were amplified from template plasmids: a backbone previously used for integrase-mediated cargo delivery in *S. elongatus* (pCargo), the Sel_Y-Int_1 *int*, and an array of synthetic *attP* sites (Twist Biosciences). The assembly was performed with NEBuilder HiFi Assembly mix (NEB, Ipswich, MA, E2621L), and transformed into Lucigen EC100D pir + cells (BioSearch Technologies, Petaluma, CA, ECP09500). Clones were sequence-verified via whole-plasmid sequencing by Quintara Biosciences.

For expression of integrases in *S. elongatus*, the broad host range lambda phage *P_R_* promoter, controlled by temperature-sensitive repressor cI857, was used; this promoter has been demonstrated for integrase expression across a diversity of bacteria [14]. Immediately downstream of *P_R_* is the *Bacteroides P_cepA_* promoter to further enhance broad-host applicability of the constructs [14]. The integrase was expressed with a strong RBS, and no terminator was explicitly included, based on cloning considerations. The nonreplicating vector on which the integrases (Sel_Y-Int_1 and the Bxb1 negative control) were delivered expresses a broad host range *aph* (kanamycin resistance) cassette, as well, under a native km^R^ promoter, the *ermF* promoter from IS4351, and the yeast *P_TEF_* promoter which has demonstrated activity in *E. coli* [14]. The *aph* gene was followed by the yeast *TEF1* terminator, and the strong bacterial rho-independent L3S2P21 terminator [30].

### Integrase testing in *P. putida* S12 and KT2440

Electrocompetent *P. putida* cells were prepared by washing subcultures (OD_600_ = 1.0) in 4°C 10% glycerol solution thrice with centrifugation steps occurring at 3000 × *g* for 15 min. All experiments were conducted from the same batch of electrocompetent cells stored at −80°C. 25 fmol of purified plasmid were electroporated (2500 V, 25 mF, 200 W, 2 mm gap cuvettes) into 50 μl of electrocompetent *P. putida* S12 or KT2440 in triplicate. Cells were recovered in LB broth for 2 h at 30°C with aeration. Eight 10-fold serial dilutions of recovered cells were plated on LB with and without tetracycline. Plates were incubated for 18 h at 30°C and colony forming units (CFU) were measured. Percent Tc^R^ was calculated by dividing the number of tetracycline resistant CFU by the total CFU detected in the absence of Tc. Recombination efficiency was calculated by dividing the percent Tc^R^ following electroporation of integrase-testing plasmids by the percent Tc^R^ following electroporation of the positive control plasmid pUCP22-*tcR*.

To screen for correct insertion of plasmid sequences at the chromosomal *attB* site, colony PCR was conducted using OneTaq DNA polymerase (NEB, Ipswich, MA, M0486S) with one primer annealing to the plasmid DNA and its pair annealing to the *P. putida* genome adjacent to amplify across the *attR* site (Supplementary Table 4) (IDT, Coralville, IA). PCR products were run on an agarose gel for visualization with an Invitrogen 1 kb plus ladder (Invitrogen, Waltham, MA, 10787018) as a size standard.

### Serial passaging

Tc^R^ colonies with integrated plasmids containing either Pal_S-Int_1 or Ppu_Y-Int_3 were cultured in LB medium at 30°C without tetracycline in triplicate. Every 24h, cultures were diluted to an OD_600_ of 0.1 in 2 ml fresh LB medium. This passage was repeated thrice, allowing for 4 days of growth in medium without tetracycline selection. Cultures were then diluted and plated to achieve single colonies on LB agar without antibiotics. A total of 72 colonies were then patched on LB agar containing tetracycline to identify the proportion of the population that had excised the integrated plasmid, and thus had lost Tc^R^.

To estimate the number of generations, the CFUs for cultures at an OD_600_ of 0.1 (0h CFU) and cultures 24 h later (24h CFU) were calculated. The number of generations (n) occurring over 24 h was calculated by *n* = [log(24h CFU) − log(0h CFU)]/log(2). This generation time was then multiplied by 4 to estimate the number of generations occurring over the 4-day period of serial passaging.

### Whole genome sequencing for insertion-site specificity

Dense Tc^R^*P. putida* S12 growth (∼100 colonies) with integrated Pal_S-Int_1, Pal_Y-Int_1, Pal_Y-Int_3, Ppu_Y-Int_1, Ppu_Y-Int_3, and Ppu_Y-Int_4 test plasmids were independently resuspended in phosphate buffered saline. Genomic DNA was then extracted from each sample using the Qiagen Blood and Tissue kit. Genomic DNA was sequenced by Plasmidsaurus (Louisville, KY) with an average coverage of 64.6×. We analyzed the sequencing by using our Readstepper software to analyze the insertional boundaries (can be found in the following repository: https://github.com/sandialabs/TIGER).

### Integrase testing in *S. elongatus*

To generate individual clones for screening integration outcomes in *S. elongatus*, recipient *S. elongatus* UTEX 2973 was inoculated from a colony and grown to saturation in 25 ml BG-11 in a flask. Donors bearing the Sel_Y-Int_1 test plasmid, and a control strain bearing the same plasmid with a Bxb1 integrase, and the HB101 helper bearing pRL443 [31] were inoculated from glycerol stocks and grown overnight with carbenicillin supplementation. The next day, cultures were washed once in LB and resuspended at an OD_600_ of 10. Each donor was mixed in a 1:1 volumetric ratio with helper. Recipient was diluted to OD_750_ = 3 in nonselective BG-11 media, mixed in a 1:1 volumetric ratio with the donor-helper mixture, and 100 μl of the mixture was spread on each of three autoclaved 25-mm HATF filters (Millipore, Darmstadt, Germany, HATF02500) on prewarmed BG-11 + 5% LB Lennox plates, for three replicates of each conjugation. Conjugations were incubated at 30°C in the dark at ambient CO_2_ overnight, then transferred to 38°C, 0.5% CO_2_, and 100 μE white light, for a total of 24 h of conjugation. Filters were then transferred to 5 ml liquid BG-11 and vortexed to lift cells. Lifted exconjugants were concentrated via centrifugation and spotted at various dilutions onto plates supplemented with kanamycin, wrapped in parafilm to retain moisture, and incubated at 38°C, 0.5% CO_2_, and 100 µE white light.

To test for integration of the Sel_Y-Int1 plasmid, kanamycin-resistant exconjugants were inoculated in 70 μl BG-11 media in a parafilm-wrapped 96-well V-bottom plate. Clones were grown for three days at 38°C, 0.5% CO_2_, and 200 μE white light, and then cultures were boiled at 65°C for 6 min followed by 95°C for 2 min. Boiled cells were subsequently screened for integration via PCR using Kapa 2G Fast master mix (Roche, Basel, Switzerland, KK5609) with primer pair OLQ1039/OLQ257 (targeting the locus with integrated plasmid) and OLQ1039/OLQ1038 (targeting the wild-type locus), cycling for 35 cycles with a 57°C annealing step and a 20s extension (Supplementary Table 4). Products were analyzed on a 1% agarose gel to determine presence or absence of integrated plasmid.

To test integration efficiency in *S. elongatus* (Supplementary Fig. 9), conjugations were performed identically, with the following modification: Three donor colonies for each donor (Sel_Y-int_1 and Bxb1) were inoculated from streakouts on LB plates supplemented with carbenicillin. After the 24-h conjugations, cells were lifted from filters in 5 ml BG-11 medium, spun to concentrate, and resuspended in in 70 μl BG-11. These were serially diluted 15:60 in BG-11, and spotted in 5 μl spots on 30-ml BG-11 or BG-11 + kanamycin plates.

### Statistical analyses

All integrase testing in *P. putida* was conducted using three biological replicates. When present, error bars on graphs denote standard deviation. For *S. elongatus* integrase efficiency testing, data was collected in biological triplicate, and to generate colonies to assay clonal integration outcomes, conjugations were performed in technical triplicate, and at least seven colonies were picked from each replicate.

## Results

### Development of IOD software

**Figure 2.**
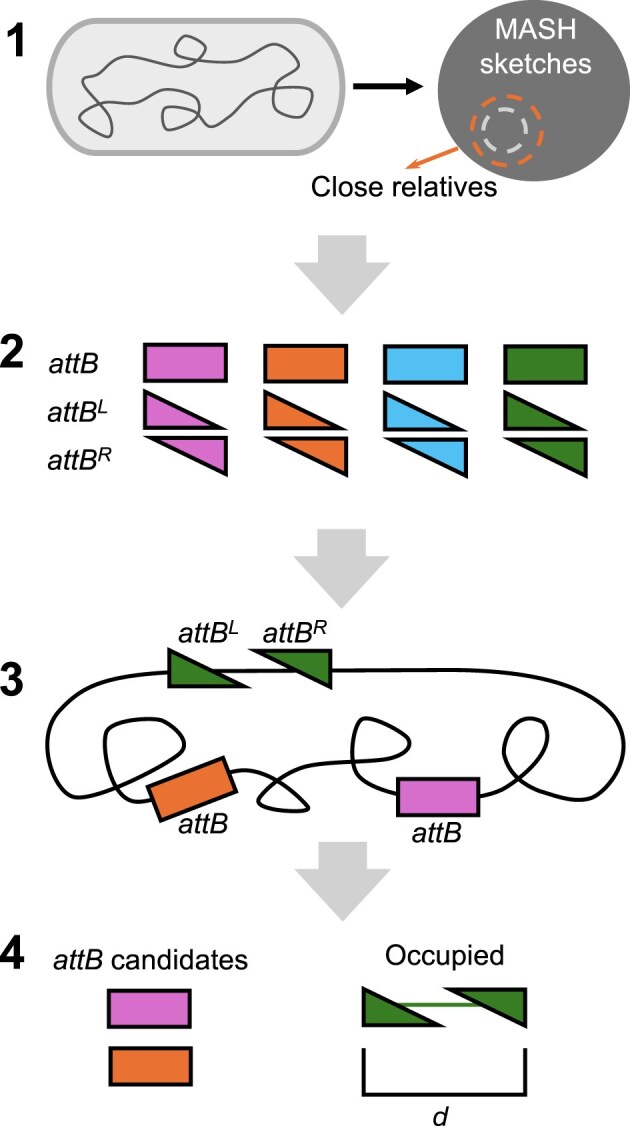
Workflow for IOD software in *taxonomic* mode. (1) MASH is used to determine close relatives of the input genome available in the IOD database. (2) A query list of *attB*s and half *attB*s (see the ‘Materials and methods’ section; represented as triangles) are used as a query against the BLASTN database of the genome of interest. (3) A series of filters locates candidate *attB* sites and collapses overlapping matches. (4) GI-occupied *attBs* (identified by distance between hits to left and right *attB halves)* are rejected and the best candidate *attB* sites in the genome are reported separately from the duplicate sites.

The IOD software identifies *attB* sites within bacterial genomes of interest to facilitate integrase-based integration of genetic cargo. We developed an *attB* database, derived from genomic data of GIs previously identified in our TIGER/Islander GI database of predicted GIs [7] resulting in a nonredundant list of 182 734 *attB* and integrase pairs. The IOD software can act on a query genome sequence in two modes: *taxonomy* mode identifies *attB* sites from close relative GIs or *search* mode which can search for *attBs* from the entire de-duplicated database (Fig. 2; see the ‘Materials and methods’ section).

*Taxonomy* mode is preferred for its computational efficiency (Supplementary Fig. 1) and biological basis. Biologically, the advantages are two-fold. (i) Taxonomically close relatives of the target bacterium are likely to exhibit similar cellular physiology and produce the same suite of integration host factors required for optimal integrase activity [32–35]. Integrase-*att* pairs output by IOD in *taxonomy* mode all function within relatives, as they have been able to insert the GI from which our sequences were sourced, increasing the likelihood that the integrase will function in the target strain. (ii) Integration events frequently interrupt coding regions, often tRNA genes [5, 8, 36]. Many *attP* sites have evolved to complete the gene that their GI is inserting within to maintain appropriate expression following integration.

To demonstrate broad potential utility of IOD for genome editing in phylogenetically diverse hosts, we have applied the IOD software to 183 genomes from a broad phylogenetic range of bacterial and archaeal species (Table 1 and Supplementary Table 1). Strains in Table 1 include many relevant for biomanufacturing or medical applications and were typically found to contain numerous candidate *attB* sites identified using IOD in *taxonomy* mode, with only three exceptions (Table 1). However, most other species we tested did not have candidate *attBs* predicted in *taxonomy* mode (97/142; Supplementary Table 1). For species in many phyla, especially those known only from environmental metagenomics, this is due to a lack of sequenced genomes from close relatives, resulting in too few *attB* search sequences. However, when running in *search* mode using all *attB* sequences in our database, only one strain out of the 183 genomes tested lacked an *attB* candidate; the total number of available *attB*s found in *taxonomic* mode was 234, rising to 1295 in *search* mode (Supplementary Table 1). There are no obvious trends as to which organisms are sources of the additional candidate *attB* sites found using *search* mode. In the cases we examined closely, source organisms span the entire phylogenetic tree with some crossing domains (e.g. integrase source is archaea and target was bacteria), further highlighting the utility of querying a large database for putative *attB* sites. These results indicate IOD software will find *attB*/integrase pairs for most bacteria and identify potential genomic locations for large DNA payload integration.

Using the 183 genomes representing diverse bacterial and archaeal phyla (Table 1 and Supplementary Table 1), we benchmarked performance speed. The average runtime per genome for both test sets was 21.93 ± 1.320 (20.61–23.25) seconds, which was not measurably influenced by genome size (0.37–16 Mbp) (Supplementary Fig. 2A). Runtime is primarily dependent on the number of *attB* sequences used to query against the genome of interest (Supplementary Fig. 2B). The maximum number of reference *attB* sequences collected from the closest relatives (based on phylogenomic comparison) of the input genome is 500 for *taxonomic* mode (run with default setting). However, there may be fewer *attB* sequences in the query depending on the species (see the ‘Materials and methods’ section), which results in a shorter runtime (e.g. *S. elongatus*). If the user runs the program in *search* mode without target sequences, the default database size is 182 734 (our entire *attB* database). The shortest allowable *attB* sequence in the database is 22 nucleotides (with default parameters). This was chosen to reduce false positives: given the probability of finding a random k-mer in a genome of length L is 2 × L × 4^−k^, the expected discovery rate for a random 22-mer in the *P. putida* S12 genome is ∼3.67 × 10^−7^, and for at least one of 500 22-mers, 1.84 × 10^−4^. Benchmarking results emphasize the value of *taxonomic* mode for *attB* sequence identification given the low expected discovery rate and the correlation between runtime and number of query *attB* sequences. However, if computational resources or time are not of concern, *search* mode may be preferable to increase to odds of identifying an integrase/*attB* pair for a target prokaryote.

### Prediction and computational analysis of IOD integrases in *P. putida*

*Pseudomonas putida* is a Gram-negative soil saprophytic bacterium that has been embraced as a biomanufacturing and synthetic biology chassis due to its hardiness, genetic tractability, and metabolic versatility [37]. To refine our software predictions, we focused on the well-established laboratory strain *P. putida* S12. We predicted 37 candidate *attB* sites using *taxonomy* mode (Supplementary Table 2) and cross-referenced with the TIGER/Islander predicted GIs (Supplementary Table 3) to verify that IOD differentiated *attB* sites from *attL/attR* sites (which already contain a GI and thus should not be provided to the user as available candidate *attB* sites). Although GI-occupied *attBs* can be utilized, the pre-existing integrases and/or excisionases may destabilize a new insert that uses its *att* sites.

The IOD software attempts to remove any spurious integrase/*attB* pairs as part of the upstream processing steps; however, additional verification may be necessary. In our initial test set for *P. putida* S12, we determined that the predicted Pmo_Y-Int_1 integrase was ∼125 aa shorter than expected [our predicted integrases had an average length of 393 aa, and the model tyrosine integrases are 356 aa (Lambda) and 343 aa (Cre)]. We developed and implemented a filter to remove short integrases (see the ‘Materials and methods’ section), filtering out 315 597 of 923 744 unique tyrosine integrase sequences and 164 205 of 353 354 unique serine integrase sequences (mainly lacking the recombinase domain) from our initial integrase/*attB* site list.

**Figure 3.**
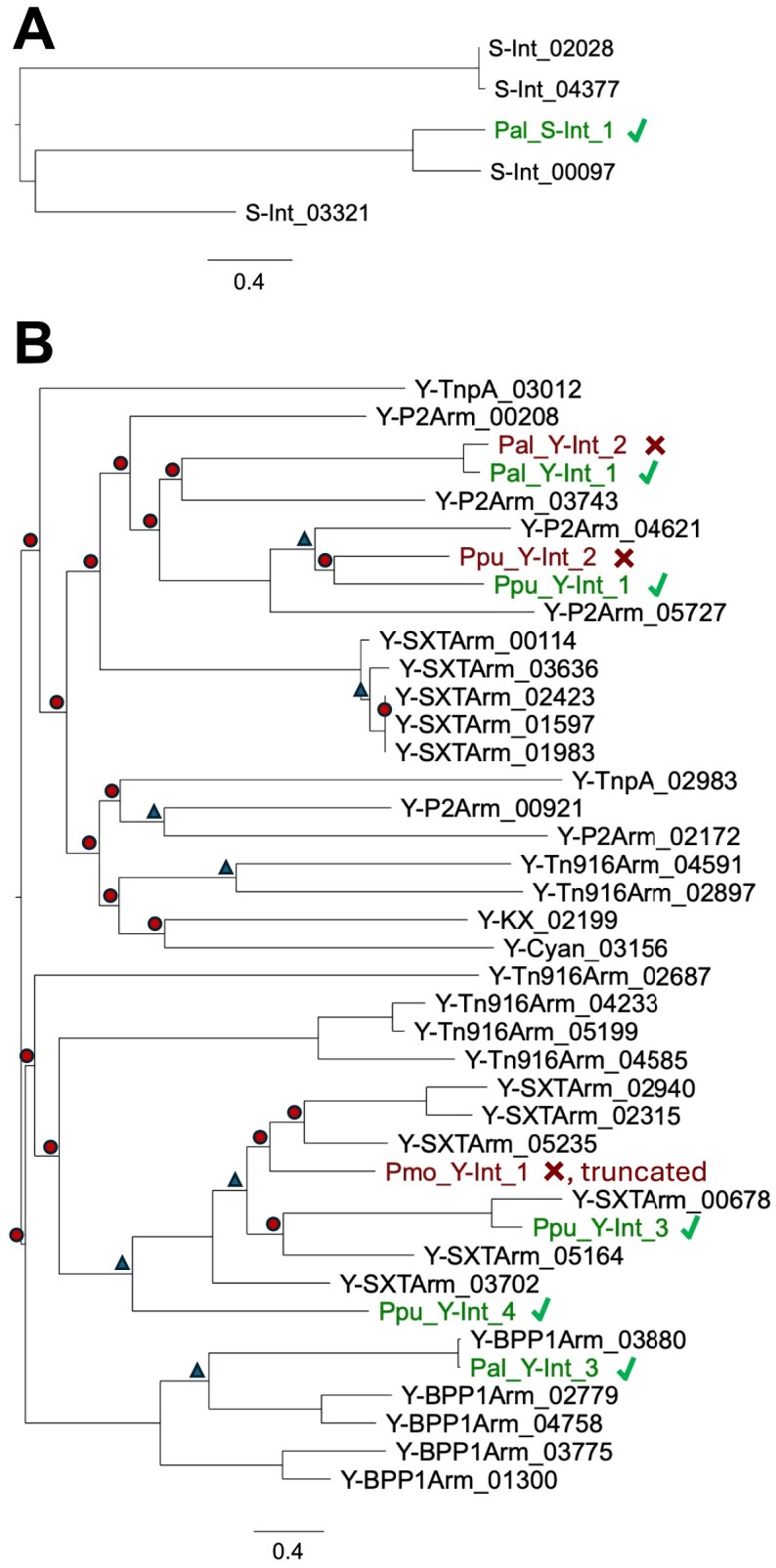
Phylogenetic tree of IOD predicted integrases for *P. putida* S12. Protein sequences were aligned to create a maximum likelihood tree for the **(A)** Serine integrases (S-) and **(B)** Tyrosine integrases (Y-). Based on results from experimental validation (Fig. 4), functional integrases are colored green and marked with a check and failed integrases are red colored with an X. Branch support values are ≥95% except for those marked with a triangle (50%–94%) or a circle (<50%).

To ensure we were testing functionally distinct integrases, we constructed two phylogenetic trees to determine the relationship between our IOD predictions for *P. putida* S12 (Fig. 3). All predicted integrases were unique, except for three (Y-STXArm_01597, Y-STXArm_01 983, Y-STXArm_02 423). We chose to experimentally validate nine integrases from across the two trees (Table 2 and Fig. 3).

**Table 2.** The nine tested integrase and *attB* pairs predicted for *P. putida* S12

Integrase name	IOD output integrase name	GI accession/coordinates	*attB* locus in *P. putida* S12
Pal_S-Int_1	S-Int_05 038	CP003588.1/5514 732–5555071	5199833–5199794
Pal_Y-Int_1	Y-BPP1Arm_00 722	JAJSPR010000024.1/13977–1 + JAJSPR010000015.1/121491–103117	3624898–3624860
Pal_Y-Int_2	Y-P2Arm_04 004	CP009974.1/4394 697–4412415	4412428–4412467
Pal_Y-Int_3	Y-P2Arm_04 082	JAJSPR010000004.1/61465–7230	4394691–4394738
Pmo_Y-Int_1	Y-SXTArm_01 031	BBIS01000052.1/21985–65155 + BBIS01000043.1/1–19 202	3480234–3480261
Ppu_Y-Int_1	Y-P2Arm_04 153	CP010979.1/4574 849–4584722	1637680–1637745
Ppu_Y-Int_2	Y-P2Arm_05 871	CP010979.1/6478 889–6483051	688725–688650
Ppu_Y-Int_3	Y-SXTArm_00 656	AP015029.1/709 926–642683	1478260–1478293
Ppu_Y-Int_4	Y-SXTArm_03 507	AP015029.1/3987 515–3844078	4394691–4394738

Details on all IOD integrase candidates are shown in Supplementary Table 2.

We evaluated the integrase protein sequences to determine whether the catalytic serine or tyrosine residues were present. Previous work on phiC31 and Bxb1 serine integrases identified essential residues for enzymatic activity; these include a conserved N-terminal domain [38], the catalytic serine (S10 in Bxb1 [39] and S12 in phiC31 [40]), and conserved residues found in the active pocket required for catalytic activity (R8, Q22, D81, R82, L83, R85 in Bxb1; R10, Q26, R93, R96 in phiC31) [41]. We confirmed that in all predicted serine integrases for *P. putida* S12, the catalytic serine residue was present, the N-terminal domain showed a high level of conservation, and the active pocket residues were fully conserved (Supplementary Fig. 3).

In tyrosine integrases, the C-terminal catalytic domain is characterized by the crucial Tyr and the Arg-His-Arg catalytic triad [42]. In all predicted tyrosine integrases, we identified the catalytic tyrosine (Supplementary Fig. 4). We observed a few mutations in the catalytic triad in the integrases in this dataset. In two integrases, the first arginine residue of the catalytic triad is mutated to a serine residue. In two integrases that were candidates for experimental validation (Pal_Y-Int_1 and Pal_Y-Int_2), the second arginine is mutated to a lysine. We also see a large clade of Y-BPP1 integrases (which includes Pal_Y-Int_3) missing the H308/H289 residue from the catalytic triad. We expect this to be a novel clade of integrases.

The lack of active recombination is an important criterion for selecting *attBs* for targeted integration because spurious excision or recombination events can reduce the stability of or damage the inserted genetic cargo. To test which predicted *attB* sites in *P. putida* S12 were optimal candidates for experimental validation of IOD, we treated *P. putida* S12 with MMC, a nonspecific DNA damaging agent, which often induces GI excision or other recombination events (potentially caused by transposons, homologous recombination, or nonhomologous end joining [43, 44]). Deep sequencing revealed that none of the *attB* sites that were selected for experimental validation in *P. putida* S12 were actively undergoing recombination (Supplementary Fig. 5). While multiple regions in the *P. putida* S12 genome have sequencing reads indicating recombination above background levels, none of these correspond to predicted GIs (Supplementary Table 3), surprisingly, suggesting that the GIs in this strain are not excised upon MMC treatment.

### Experimental validation of predicted integrases for *P. putida S12*

**Figure 4.**
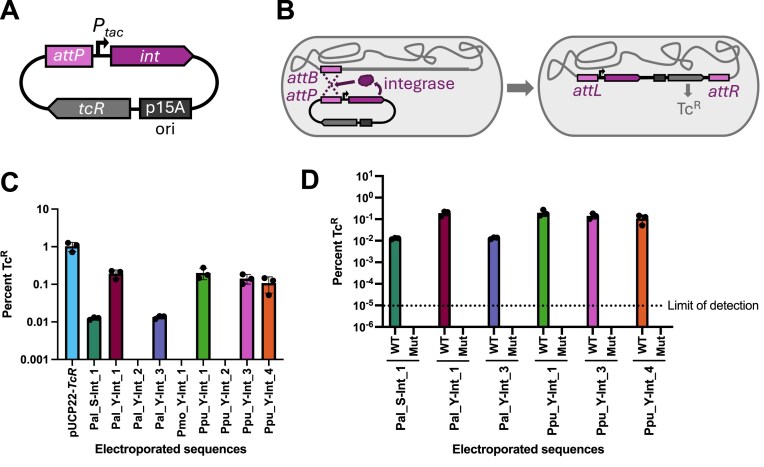
Using a nonreplicating vector-based approach, 6/9 tested integrase and *attP* pairs mediate recombination in an integrase and *attP* sequence-specific manner. **(A)** Diagram of an integrase testing plasmid. The testing plasmids bear the integrase gene (*int*) driven by a *P_tac_* promoter, the 600 bp cognate *attP* sequence, a tetracycline resistance-conferring gene (*tcR*), and an origin that will not replicate in *Pseudomonas* (p15A ori). **(B)** Schematic of integrase-mediated recombination between *attP* and *attB* sites that integrates the integrase testing plasmid sequences into the chromosome, conferring stable tetracycline resistance. **(C)** Percent of the population with tetracycline resistance (Tc^R^) following electroporation of 25 fmol of an integrase test plasmid bearing the *int* and cognate *attP* of the listed integrase or a positive control, replicating plasmid, pUCP22-*TcR*. The limit of detection was 10^−5^% TcR cells. **(D)** Percent of the population with tetracycline resistance (Tc^R^) following electroporation of 25 fmol of wildtype (WT) a functional integrase test plasmid or (Mut) a plasmid containing either an incorrect *attP* sequence (Pal_Y-Int_1, Ppu_Y-Int_1, Ppu_Y-Int_3, Ppu_Y-Int_4) or a frameshifted *int* (Pal_S-Int_1, Pal_Y-Int_3). Values reported in panels (C) and (D) are raw numbers and not normalized.

We constructed tetracycline resistance-conferring, nonreplicating vectors containing 600 bp *attP* sequences (Supplementary Fig. 6) and the *tac* promoter driving expression of the cognate integrase (*int*) gene (Fig. 4A and Supplementary Table 5). Because the vector does not replicate in *Pseudomonas*, plasmids that bear sequences encoding a nonfunctional integrase will be unable to recombine into the chromosome and thus the plasmid, and the conferred tetracycline resistance, will be diluted out of the population. If the *int* sequence encodes a functional integrase and the predicted *attB* and *attP* are correct, the protein should mediate recombination between the plasmid *attP* sequence and the chromosomal *attB* sequence, stably conferring tetracycline resistance (Tc^R^) (Fig. 4B).

Electroporation of plasmids bearing integrases Pal_S-Int_1, Pal_Y-Int_1, Pal_Y-Int_3, Ppu_Y-Int_1, Ppu_Y-Int_3, and Ppu_Y-Int_4 yielded an average of 0.01%, 0.19%, 0.01%, 0.20%, 0.14%, and 0.11%, Tc^R^ cells respectively (calculated by Tc^R^ CFU/total CFU) (Fig. 4C). Therefore, these six integrases and *attP* pairs identified by the IOD software are capable of facilitating DNA recombination in *P. putida* S12. As expected, the heavily truncated Pmo_Y-Int_1 failed to yield Tc^R^ colonies, as did two additional integrases, Pal_Y-Int_2, and Ppu_Y-Int_2, indicating that these three predicted integrases could not mediate recombination between the identified *att* sites in our assay (Fig. 4C).

Because acquiring stable Tc^R^ relies on successful electroporation followed by integrase-mediated recombination, to calculate integration efficiency, we must account for electroporation efficiency. To assess the rate of plasmid uptake through electroporation for our population, we generated pUCP22-*tcR*, a tetracycline-resistance conferring, replicating plasmid that is similar in size to the *P. putida* S12 integrase-testing plasmids. Electroporation of 25 fmol of this plasmid yielded a population with 1.02% Tc^R^ cells, indicating that in our assay, electroporation will deliver a plasmid of nearly 6100 kb to 1/100 cells in the population (Fig. 4C). At this electroporation efficiency, integrases Pal_S-Int_1, Pal_Y-Int_1, Pal_Y-Int_3, Ppu_Y-Int_1, Ppu_Y-Int_3, and Ppu_Y-Int_4 facilitated recombination in an average of 1.2%, 18.7%, 1.3%, 19.4%, 13.8%, and 10.5% of the cells that received the plasmid (Supplementary Fig. 7).

**Figure 5.**
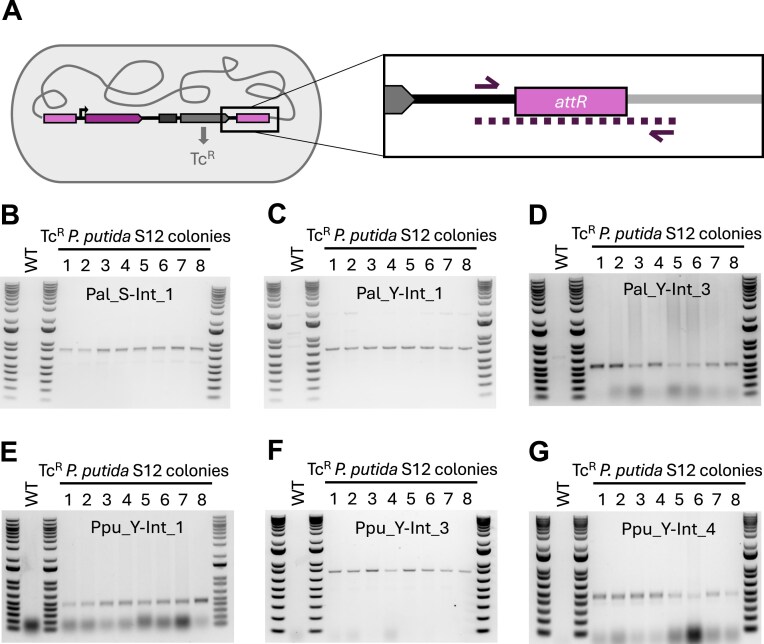
Functional integrases mediate site-specific recombination between predicted *attB* sites. **(A)** To verify recombination occurs at the predicted chromosomal *attB* and plasmid *attP* sites, DNA from *attR*-containing junctions were PCR amplified using a primer annealing to chromosomal DNA and a primer annealing to plasmid DNA. **(B–G)** PCR products using wild-type *P. putida* S12 (WT) or Tc^R^*P. putida* S12 colonies post electroporation of *int* genes encoding **(B)** Pal_S-Int_1, **(C)** Pal_Y-Int_1, **(D)** Pal_Y-Int_3, **(E)** Ppu_Y-Int_1, **(F)** Pal_S-Int_1, **(G)** Ppu_Y-Int_4, and cognate *attP* containing test plasmids.

To verify that plasmid insertion into the chromosome is dependent on integrase-mediated recombination at *attB* sites and not mediated by other modes of recombination, we created additional mutant plasmids with either (i) single nucleotide deletions in the *int* sequence to produce frameshifted, nonfunctional integrase proteins or (ii) false *attP* sequences (see the ‘Materials and methods’ section). We observed that mutated versions of functional integrase/*attP* pairs did not generate Tc^R^ cells, and thus, we conclude that recombination is dependent on integrase activity and *attP* site sequence specificity (Fig. 4D). To further verify that insertion is *attB* sequence-specific, we PCR amplified *attR* regions using a primer annealing to the plasmid backbone and a primer annealing to the chromosome (Fig. 5A). For each integrase that mediated recombination, 8/8 screened Tc^R^ colonies yielded PCR products of the size expected for integration within the *attB* site, and no amplification was observed in the wild-type *P. putida* S12 negative control (Fig. 5B–G). To further probe for off-site genomic insertions, we conducted whole genome sequencing on mixed populations of Tc^R^ cells (∼100 colonies growing on a tetracycline-supplemented plate), following independent electroporation of the six functional integrase test plasmids. Whole genome sequencing for all tested integrases indicated that integration occurred at the predicted *attB* site, forming the expected *attR* and *attL* sites, with no evidence of off-target insertions. Together, these results indicate that the tested integrases are mediating recombination in an *attB* site-specific manner. Interestingly, tandem copies of inserted plasmids were present in a small amount of sequencing reads mapping to the test plasmid (1%) within half of the samples. These duplicate copies could be created by integration at a reconstituted *attB* site following successful integration of the first copy or by homologous recombination between the >6 kb of sequence homology shared between an integrated construct and an incoming plasmid.

Integrase-inserted gene cargo should remain stably integrated when introduced unless cognate excisionases are present to allow the reverse reaction to occur or there are other sources of genome instability. To test the stability of our integrated constructs, we serially passaged *P. putida* S12 with two integrated cargos (Pal_S-Int_1 and Ppu_Y-Int_3) in antibiotic-free medium every 24 h for 4 days. By comparing growth on LB with and without tetracycline supplementation, we observed that the Pal_S-Int_1 test cargo remained stably integrated in the genome (0% Tc^S^), while the Ppu_Y-Int_3 test cargo excised in a small proportion of cells (1.39% Tc^S^) over approximately 26 generations. Excision may be due to induction of a cross-reactive excisionase (five putative excisionases and 22 integrases are annotated in *P. putida* S12), or if the integrase is capable itself of catalyzing excision (i.e. it is bidirectional), or other processes such as endogenous recombination. Depending on the cause of mobility, one can improve stability by utilizing integrases experimentally verified to mediate stable insertion or maintaining (e.g. antibiotic) selection pressure. Cross-reacting excisionases already present in the target genome can be addressed by selecting an alternative integrase or curing the strain of the offending interfering GI. Bidirectional integrases can also be identified experimentally and excluded, or be tamed by providing the *int* on a separate nonintegrating plasmid, or genetically inactivating the *int* once the construct is integrated.

We also used IOD to identify integrase and *attB* pairs for *P. putida* strain KT2440 (Supplementary Table 2), which is used more frequently than *P. putida* S12 in biomanufacturing applications [37]. We observed that three of the six functional integrases tested in S12 were also predicted by the IOD software for KT2440 (Pal_Y-Int_1, Ppu_Y-Int_1, and Ppu_Y-Int_3; Supplementary Table S2). Using the same integrase test plasmids and assay, we demonstrated that all three integrases facilitated recombination in a sequence-specific manner, as evident from their generation of stably Tc^R^ colonies at 1.14%, 2.43%, and 2.39% of the total population respectively, and an absence of colonies for the controls lacking integrase activity (Supplementary Fig. 8A and B).

### Experimental validation of a predicted integrase in *S. elongatus*

**Figure 6.**
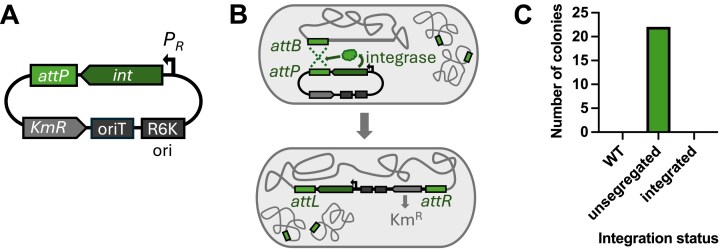
Testing Sel_Y-Int_1 integration at native cyanobacterial *S. elongatus attB* site. **(A)** Schematic of the kanamycin resistance-conferring, nonreplicating plasmid bearing an R6K ori, origin of transfer for conjugation (oriT), the Sel_Y-Int_1-encoding *int* driven by the lambda phage pR promoter, and the cognate *attP* site. **(B)** Schematic of integrase-mediated recombination between the plasmid *attP* site and the many copies of the chromosomal *attB* site, which leads to stable kanamycin resistance. **(C)** Km^R^ clones obtained were assayed for integration via PCR of the *attB* site. “Integrated” clones would have yielded only a band detecting the integrated plasmid, whereas “unsegregated” clones displayed both wild-type “WT” and integrated loci. WT clones would have displayed amplification for only the undisrupted locus, but no such instances were detected.

To further demonstrate the potential utility of the IOD method, we sought to test a predicted integrase and *attP* pair for site-directed chromosomal integration in a cyanobacterial strain, *S. elongatus* UTEX 2973. Many cyanobacteria are notoriously difficult to engineer, due to their high copy number of chromosomes per cell [45, 46], and slow chromosome segregation rates, so tools for efficient genomic integration are valuable. We applied our IOD software to *S. elongatus* UTEX 2973 in *taxonomy* mode and discovered a single integrase and *attB* pair (Supplementary Table 2). To test this integrase in our target strain, we conjugated *S. elongatus* with a nonreplicating vector bearing the lambda phage pR promoter-driven *int* encoding the Sel_Y-Int_1 integrase, *attP* site, and a kanamycin resistance marker to select for integrants (Fig. 6A and B). To control for nonspecific integration, we also conjugated the same construct with the Sel_Y-int_1 *int* replaced with the Bxb1 *int*, whose *attB* site is not found in the *S. elongatus* genome. Exconjugants from Sel_Y-int_1 grew on selective media, while the Bxb1 exconjugants did not (Supplementary Fig. 9), indicating that survival of the Sel_Y-int_1 transformants was mediated by Sel_Y-int_1 activity towards its native *attB* site. Spotting of serial dilutions of exconjugants suggested that integration efficiencies were ∼1%, but due to the density dependence of the cyanobacterial growth on selective media, precise integration efficiencies could not be ascertained (Supplementary Fig. 9). To confirm that selected exconjugants had undergone integration of the test plasmid, we screened kanamycin-resistant (Km^R^) colonies for integration at the native *attB* site via PCR. Because *S. elongatus* bears numerous chromosome copies, successful Sel_Y-Int_1-mediated recombination may occur at one or multiple *attB* loci in a single cell, resulting in a colony with a mixture of integrated and wild-type chromosome copies (“unsegregated”). All tested colonies demonstrated integration in some chromosome copies, and in most colonies, WT bands were very faint, suggesting almost complete segregation at the locus (Fig. 6C and Supplementary Fig. 10).

## Discussion

Here, we have introduced IOD—a method for computationally identifying and experimentally utilizing native *attB* sites and their cognate integrases for genomic insertion of large DNA cargo. We have experimentally validated seven predictions in three biotechnologically relevant strains of bacteria (*P. putida* S12 and KT2440 and *S. elongatus* UTEX 2973), but IOD can be extended to a wide range of prokaryotes. These results illustrate the utility of our IOD method across phyla, and demonstrate use of both electroporation (*P. putida*) and conjugation methods (*S. elongatus*) for delivery of genetic cargo with these discovered integrases. Further, we tested integration of relatively small cargo sizes (∼6 kb in *P. putida* and 11 kb in *S. elongatus*); however, based on the size of naturally occurring GIs, we believe it likely that much larger cargos could generally be delivered using IOD-predicted integrases [12, 47].

IOD also provides access of integrase-mediated genetic editing to a diversity of nonmodel microbes. We predicted open *attB* sites for all but one strain when we applied our IOD software to genomic sequences from 183 strains of bacteria and archaea spanning the tree of life (182/183 tested). The IOD software can search for candidate *attB* sites in any genomic sequence, including eukaryotes, though successes for nonprokaryotes may be infrequent due to low sequence similarity with the prokaryote-sourced *attB* sites. Furthermore, utilizing integrase/*att* pairs that are drawn from genomes of near phylogenetic relatives to the strain of interest are less likely to require host factors or host background conditions not present in strain of interest. Lastly, while the IOD method entails using the endogenous *attB* sites for genome editing, the IOD database significantly increases the number of known integrase/*attB* pairs. This database could be mined to diversify integrases/*att* pairs used in other integrase-based technology such as SAGE or PASTE.

Integrases mediate stable DNA integration, but the plasticity of prokaryotic genomes may lead to loss of the inserted DNA cargo from the population over time. Our software aids in improving integration site-specificity, stability, and efficiency by filtering out *attB* sites present at multiple copies, those within GIs, and those whose predicted integrase appears to be defective in some way. While we performed numerous follow up experiments in *P. putida* to ensure our *attB*/integrase pairs were high quality, this step is not required and was not done for *S. elongatus*. However, researchers who want to further ensure that IOD integration is site-specific and stable may exclude *attB* sites that fall within or close to experimentally identified recombination hotspots [29]. Additionally, promiscuous native integrases present in the target strain could, if actively produced, be cross reactive with the selected IOD *attP*. We suggest users compare amino acid sequences to ensure that the chosen IOD integrase is distinct from native integrases in the target strain. Finally, stability can be enforced by maintaing antibiotic selection to ensure that target cell populations maintain the inserted cargo. Future software improvements may include versions flagging integrases in site-promiscuous clades and those which likely disrupt essential genes (e.g. developmental genes [5]), to further improve output candidate integrases and *attB* pairs for genomic integration. However, systematic studies are required to establish reliable and efficient criteria for these searches.

By utilizing *attB* sites native to a host, IOD bypasses the requirement for multiple transformations or insertion of *attB* landing pads that are needed for traditional integrase-mediated genomic integration approaches [15–17]. While integration into the endogenous *attB* sites may cause fitness defects, this is unlikely for a number of reasons. Many GIs restore the gene they insert into producing a functional copy that reduces any impact on bacterial physiology [5, 8]. The GIs that utilize these *attB* sites have persisted in bacterial genomes throughout evolutionary history [48], suggesting the fitness impact is positive or neutral for many GIs that use these sites. In most cases, synthetic integration cassettes that utilize the naturally occurring *att* sites predicted by IOD software can be designed to emulate the genetic neutrality of the natural GIs from which these originate. Further, as has been done with landing pad *att* sites, these endogenous *att* sites can be tested in genetically tractable organisms, established as being correct matches for the integrase, and integrase and *att* sites used in any organism in which the *attB* sites are found.

Serine integrases have traditionally been favored for biotechnological applications, due to their reduced requirements (e.g. short *att* sequences and no required cofactors) and greater integration stability compared to tyrosine integrases [11]. However, our test cases demonstrate that the tyrosine integrases also have great general utility, mediating genomic integration at comparable rates to the tested serine integrase. Utilizing tyrosine integrases significantly broadens the applicability of IOD, because tyrosine integrases are far more abundant in prokaryotic genomes than serine integrases (∼8-fold), which greatly expands the potential *attB* sites that may be present within a target organism. Furthermore, IOD enables selection of integrases from close relatives of a target strain, mitigating any potential dependencies that the integrases might have on host factors. Because of this, IOD extends the possibility of genetic engineering to virtually all prokaryotes, including those in microbiomes, to facilitate site-specific delivery of large cargo into the genome. Finally, we note that our results have been produced using *att* site sequences and integrase sequences exactly as found in nature, without optimization. Further work may enable increased integration efficiency, shortening and simplifying att sites, and use of *att* site sequence variation to increase species coverage.

## Supplementary Material

gkag106_Supplemental_Files

## Data Availability

All data underlying this article are available in the article or in the online supplementary material. All genomes processed are available at NCBI GenBank and the genome accession numbers are listed in Supplementary Table 1, in the GCA column (search for GCA_the 9 digit number at NCBI, https://www.ncbi.nlm.nih.gov/). The software is freely available at https://github.com/sandialabs/Integrase-On-Demand. We have deposited the IOD software used for this analysis at zenodo under this DOI: 10.5281/zenodo.17576197. Readers can find the underlying databases at this DOI: 10.5281/zenodo.17088441.
